# Supplementary material for: The metal binding site composition of the cation diffusion facilitator protein MamM cytoplasmic domain impacts its metal responsivity
Source: Sci Rep. 2020 Aug 20;10:14022. doi: 10.1038/s41598-020-71036-4 (PMC7441159; doi:10.1038/s41598-020-71036-4)
Supplement: Supplementary file 1 [file 41598_2020_71036_MOESM1_ESM.pdf]

Supplementary information for:

The metal binding site composition of the cation diffusion facilitator protein MamM cytoplasmic domain impacts its metal responsivity

Authors: Shiran Barber-Zucker[1,2,3], Anat Shahar[2], Sofiya Kolusheva[3] and Raz Zarivach[1,2,3,*]

[1] Department of Life Sciences, Ben-Gurion University of the Negev, Beer Sheva 8410501, Israel

[2] The National Institute for Biotechnology in the Negev, Ben-Gurion University of the Negev, Beer Sheva 8410501, Israel

[3] Ilse Katz Institute for Nanoscale Science and Technology, Ben-Gurion University of the Negev, Beer Sheva 8410501, Israel

* Correspondence should be addressed to Raz Zarivach, Department of Life Sciences, Ben-Gurion University of the Negev, P.O.B. 653, Beer Sheva 8410501, Israel. Tel: +972-8-6461999; Fax: +972-8-6472970; Email: zarivach@bgu.ac.il

Supplementary Results and Discussion

In the main text we focused on the analyses of the differences between the ability of Zn^{2+}, Ni^{2+} and Mn^{2+} to be bound by different mutants (see main text *Results and Discussion*). Here we give detailed analyses of Cu^{2+} and Cd^{2+} binding to the varied mutants.

<u>MamM CTD mutants binding to Cu^{2+}:</u> For this study we solved the crystal structures of MamM CTD D249H and D249N with Cu^{2+}, while we previously solved the structure of the Cu^{2+}-bound WT CTD[1]. The WT bound structure shows tighter conformation compared to the WT apo form (Figure S5A), with Cu^{2+} ions bound in the central binding site (one ion by H285 from both monomers) and peripheral binding sites (in each site, one ion by H264 from one monomer and a water molecule that bridges the E289 residue from the second monomer) (Figure S5B). Both D249H and D249N Cu^{2+}-bound structures exhibit the same conformation as the WT apo form, with the Cu^{2+} only bound by the H264 residues and water molecules (Figure S5C). Although in the WT bound structure the D249 does not participate in the chelation of the Cu^{2+} ions, it appears from the D249H and D249N Cu^{2+}-bound structures that this residue is important for the correct metal binding and conformational change. The bound Cu^{2+} by H264 in the D249N/H structures again hint at the importance of this residue for the chelation of the Cu^{2+} ions. Even if the H264 binding does not lead to conformational changes, it might act as an intermediate binding site. This is also supported by the Trp-fluorescence results of H264E, that show the least quenching of all mutants. In contrast to the crystal structures, the Trp-fluorescence results of the WT and the D249N/H mutants (Figure 2E, lower panel) show that D249H has similar quenching to the WT, while the D249N exhibits much less quenching. Hence, the asparagine in position 249 seems to inhibit the binding to the H285 residues, thereby causing less quenching, while the histidine does not. This suggests that in the case of D249H, the crystal structure does not accurately represent the state in solution. Cu^{2+} was shown to be bound almost exclusively by histidine in crystal structures[2], and in the WT Cu^{2+}-bound structure it also appeared that the chelation by the histidine residues mostly contributes to the conformation stability. Hence, we propose that in contrast to the N249 residue in the D249N mutants, the H249 residue contributes to the stabilization of the Cu^{2+} binding in the central site and to the closed conformation, although not observed in the crystal structure. The Trp-fluorescence results of D249H-H285D show also the same quenching pattern as in the WT and D249H, suggesting that indeed a histidine residue in the central site is important for proper chelation and conformational change.

<u>MamM CTD mutants binding to Cd^{2+}:</u> For this study we solved the crystal structures of MamM CTD D249H, D249E, D249N, H285D, H264E (two different SGs), H264E-E285H and E289D with Cd^{2+}; we previously solved the structure of the Cd^{2+}-bound WT CTD[1]. Only two Cd^{2+}-bound structures exhibit tighter conformation than the WT apo structure; one of the H264E structures and E289D (Figure S6A). Both structures exhibit the same conformation, but different binding site. While the H264E structure exhibits the same binding site as the H264E Zn^{2+}-bound structure (H285 from one monomer and H236 and E289 from the second monomer), the E289D exhibits a unique Cd^{2+}-pair binding site, as

discussed in detail in the main text. All other MamM CTD constructs with Cd^{2+}, including the WT, adopt the same conformation as the WT apo, and the Cd^{2+} is bound only in the central binding site by the residues in positions 249, 285 or both (Figure S6B). The exact Cd^{2+} coordination depends on the substitution identity. In most mutants there is only one Cd^{2+} ion bound to each monomer, while in others (WT and H264E) there are two ions that populate the binding site, but in half occupancy, suggesting that only one ion is effectively bound in them as well, by one of either binding sites. Furthermore, in some mutants the Cd^{2+} is chelated by H213 and/or E215 from a non-biological monomer from an adjacent unit, which helps to form stable coordination of the Cd^{2+} ions in the crystal form. Interestingly, in all structures where H285 residue exists it is involved in the chelation of the Cd^{2+} ions, except in D249H where the H249 rather than H285 residue binds the ion. In the H285D mutant, the aspartate at position 249 binds the ion, overall showing that both the 249 and 285 positions and both histidine and aspartate residues can bind the Cd^{2+} ions effectively. Previous PELDOR results show that the WT CTD binding to Cd^{2+} lead to a tight rigid conformation, similar to that of the WT CTD with Zn^{2+} and Cu^{2+}, and in contrast to the Cd^{2+}-bound crystal structure[1]. This indicates that for Cd^{2+}, the crystal structures do not necessarily represent well the structures in solution. In some mutants, Cd^{2+} binding might not lead to conformational changes (as discussed below). In mutants that do no exhibit conformational changes in the Cd^{2+}-bound structures but do in solution, it is possible that Cd^{2+} binding to each monomer prevents the tighter association of the monomers due to the large size of the Cd^{2+} ions. Such an ion would not afford correct movement and stable tight conformation due to charge repulsion and steric interference. When considering the Trp-fluorescence results (Figure 2B, top panel), one can see that in solution the conformational changes occur, with each construct exhibiting different conformation. For example, H264E shows no blue-shift but one of its crystal forms showed tighter conformation, suggesting that H264E Cd^{2+}-bound structure, that exhibits no conformational change, is the conformation in solution (as discussed in the main text). E289D, whose crystal structure shows tighter conformation, shows a smaller blue shift compared to the WT. In this case, since: (1) The E289D-Cd^{2+} and the H264E-Zn^{2+} structures exhibit similar dimerization, (2) The addition of Cd^{2+} to E289D and the addition of Zn^{2+} to H264E result in smaller blue-shift as compared to their addition to the WT CTD, and (3) Zn^{2+}-bound and Cd^{2+}-bound conformations in solution are similar for the WT CTD (as evident by PELDOR[1]), we propose that the crystal structure of E289D-Cd^{2+} also represents well the structure in solution. D249H shows the largest blue-shift, suggesting that the binding to position 249 exclusively (this is the only form where the ion was bound only at this position) impacts more on the conformational changes or on the Trp signal (as position 249 is closer to W247 than position 285).

Supplementary Tables

Table S1: Crystallization of MamM CTD constructs with different metals.

Protein name	**H264E**	**H264E – Zn^{2+} form1**	**H264E – Zn^{2+} form2**	**H264E – Cd^{2+} form1**	**H264E – Cd^{2+} form2**
PDB code	6H5V	6H5M	6H5U	6H8G	6HAO
Crystallization conditions	0.2M $(NH_4)_2SO_4$, 0.1M BIS-TRIS pH=5.5, 25% PEG 3350	0.5M $Mg(HCO_2)_2$, 0.1M HEPES pH=7.5	0.2M $(NH_4)_2SO_4$, 0.1M BIS-TRIS pH=6.5, 25% PEG 3350	0.2M $(NH_4)_2SO_4$, 0.1M BIS-TRIS pH=5.5, 25% PEG 3350	0.2M $MgCl_2$, 0.1M BIS-TRIS pH=6.5, 25% PEG 3350
Metal salt used (3.375 mM)	$MnCl_2$	$ZnCl_2$	$ZnCl_2$	$CdCl_2$	$CdCl_2$
Cryo protectant	50% PEG 3350	60% crystallization condition, 15% MQ, 25% ethylene glycol	50% PEG 3350	-	50% PEG 3350
Data collection	ESRF – ID30B	ESRF – ID30B	ESRF – ID30B	ESRF – ID29	BESSY II – BL 14.1
Detector	Pilatus3 6M	Pilatus3 6M	Pilatus3 6M	Pilatus 6M	Pilatus 6M

Protein name	**D249N**	**D249N – Zn^{2+} form**	**D249N – Cd^{2+} form**	**D249N – Cu^{2+} form**	**D249N – Ni^{2+} form**
PDB code	6H88	6H87	6H8A	6H89	6H8D
Crystallization conditions	0.1M HEPES pH=7.5, 25% PEG 3350	0.3M $Mg(HCO_2)_2$,0.1M Tris pH=8.5	0.2M Li_2SO_4, 0.1M BIS-TRIS pH=5.5, 25% PEG 3350	0.2M $(NH_4)_2SO_4$, 0.1M BIS-TRIS pH=5.5, 25% PEG 3350	0.2M Li_2SO_4, 0.1M BIS-TRIS pH=5.5, 25% PEG 3350
Metal salt used (3.375 mM)	$MnCl_2$	$ZnCl_2$	$CdCl_2$	$CuSO_4$	$NiCl_2$
Cryo protectant	50% PEG 3350	50% PEG 3350	50% PEG 3350	-	-
Data collection	ESRF – ID29	ESRF – ID29	ESRF – ID29	ESRF – ID29	ESRF – ID29
Detector	Pilatus 6M	Pilatus 6M	Pilatus 6M	Pilatus 6M	Pilatus 6M

Protein name	**D249E – Zn^{2+} form**	**D249E – Cd^{2+} form**	**D249H – Cd^{2+} form**	**D249H – Cu^{2+} form**	**D249H-H285D**
PDB code	6H5K	6H9Q	6H84	6H83	6HA2
Crystallization conditions	0.3M $Mg(HCO_2)_2$,0.1M Tris pH=8.5	0.2M Li_2SO_4, 0.1M BIS-TRIS pH=5.5, 25% PEG 3350	0.2M $(NH_4)_2SO_4$, 0.1M BIS-TRIS pH=5.5, 25% PEG 3350	0.2M $(NH_4)_2SO_4$, 0.1M BIS-TRIS pH=5.5, 25% PEG 3350	0.1M NaOAc, pH=4.5, 25% PEG 3350
Metal salt used (3.375 mM)	$ZnCl_2$	$CdCl_2$	$CdCl_2$	$CuSO_4$	$MnCl_2$
Cryo protectant	60% crystallization condition, 15% MQ, 25% ethylene glycol	-	-	50% PEG 3350	50% PEG 3350
Data collection	ESRF – ID30B	ESRF – ID29	ESRF – ID23-1	ESRF – ID23-1	BESSY II – BL 14.1
Detector	Pilatus3 6M	Pilatus 6M	Pilatus 6M	Pilatus 6M	Pilatus 6M

Protein name	**H285D**	**H285D – Cd^{2+} form**	**E289H – Ni^{2+} form**	**H264E-E289H**	**H264E-E289H – Cd^{2+} form**
PDB code	6H8I	6H9T	6H81	6HAN	6H85
Crystallization conditions	0.2M $(NH_4)_2SO_4$, 0.1M BIS-TRIS pH=5.5, 25% PEG 3350	0.2M Li_2SO_4, 0.1M BIS-TRIS pH=5.5, 25% PEG 3350	0.2M $(NH_4)_2SO_4$, 0.1M BIS-TRIS pH=5.5, 25% PEG 3350	0.2M $(NH_4)_2SO_4$,0.1M HEPES pH=7.5, 25% PEG 3350	0.2M $(NH_4)_2SO_4$, 0.1M BIS-TRIS pH=5.5, 25% PEG 3350
Metal salt used (3.375 mM)	$MnCl_2$	$CdCl_2$	$NiCl_2$	$MnCl_2$	$CdCl_2$
Cryo protectant	50% PEG 3350	50% PEG 3350	50% PEG 3350	50% PEG 3350	50% PEG 3350
Data collection	ESRF – ID29	ESRF – ID29	ESRF – ID23-1	BESSY II – BL 14.1	DLS – I04
Detector	Pilatus 6M	Pilatus 6M	Pilatus 6M	Pilatus 6M	Pilatus 6M-F

Protein name	**E289D – Mn^{2+} form**	**E289D – Cd^{2+} form**
PDB code	6H9P	6HHS
Crystallization conditions	0.2M Li_2SO_4, 0.1M BIS-TRIS pH=5.5, 25% PEG 3350	0.1M $(NH_4)_2SO_4$, 0.1M BIS-TRIS pH=6.5, 0.1M NaCl
Metal salt used (3.375 mM)	$MnCl_2$	$CdCl_2$
Cryo protectant	50% PEG 3350	-
Data collection	ESRF – ID29	BESSY II – BL 14.1
Detector	Pilatus 6M	Pilatus 6M

Table S2: Data collection and refinement statistics of MamM CTD constructs with different metals.

Protein name	**H264E**	**H264E – Zn^{2+} form1**	**H264E – Zn^{2+} form2**	**H264E – Cd^{2+} form1**	**H264E – Cd^{2+} form2 [a]**
PDB code	6H5V	6H5M	6H5U	6H8G	6HAO
Data collection	ESRF – ID30B	ESRF – ID30B	ESRF – ID30B	ESRF – ID29	BESSY II – BL 14.1
Space group	C 2 2 2_1	P 3_1 2 1	C 1 2 1	C 2 2 2_1	P 3_1 2 1
Cell dimensions					
a, b, c (Å)	37.18, 94.37, 53.09	53.27, 53.27, 61.42	88.05, 55.65, 63.77	37.00, 94.50, 53.21	53.66, 53.66, 58.07
α, β, γ (°)	90, 90, 90	90, 90, 120	90, 92.14, 90	90, 90, 90	90, 90, 120
Resolution (Å)	1.49-47.18 (1.49-1.52)	1.60-46.14 (1.60-1.63)	2.00-47.03 (2.00-2.05)	1.35-47.25 (1.35-1.37)	2.40-46.47 [b] (2.40-2.49)
Rsym or Rmerge	0.065 (2.454)	0.061 (1.898)	0.083 (0.146)	0.037 (1.443)	0.169 (0.463)
I/σI	13.9 (0.7)	17.5 (1.3)	23.2 (13.9)	22.5 (1.3)	11.9 (2.3)
$CC_{1/2}$	0.998 (0.356)	0.999 (0.640)	0.997 (0.993)	0.999 (0.575)	0.994 (0.910)
Completeness (%)	99.6 (94.5)	99.7 (92.3)	99.8 (96.3)	99.9 (99.1)	98.6 (88.4)
Redundancy	8.6 (7.5)	12.7 (11.5)	11.8 (12.0)	7.1 (7.1)	9.2 (4.7)
Wavelength (Å)	0.97624	0.97624	0.97624	0.976247	0.9184
No. unique reflections	15616	13738	20715	20888	3988
Refinement					
Resolution (Å)	1.49-47.18	1.60-46.14	2.00-47.03	1.35-47.25	2.40-46.47
Rwork/Rfree	19.97/22.88	21.22/25.52	17.03/22.05	16.63/19.22	23.76/31.44
No. atoms					
Protein	644	688	A-712, B-700, C-647, H-61	666	714
Ligand/ion	10	5	25	17	5
Water	49	25	159	53	11
B-factors					
Protein	35.11	43.25	A-28.959, B-28.211, C-31.400, H-40.205	26.59	45.75
Ligand/ion	SO_4^{2-}-35.49, βME-63.63, Cl^--83.24	Zn^{2+}-29.98, βME-55.92	SO_4^{2-}-50.76, Zn^{2+}-16.44, βME-44.31	SO_4^{2-}-38.18, Cd^{2+}-44.40, βME-51.75	Cd^{2+}-32.92, βME-39.58
Water	40.42	45.85	33.44	38.82	33.00
RMSD					
Bond lengths (Å)	0.010	0.013	0.014	0.014	0.005
Bond angles (°)	1.437	1.617	1.467	1.624	0.900

Ramachandran statistics [c]	P: 75 (97.40%), A: 1 (1.30%), O: 1 (1.30%)	P: 86 (98.85%), A: 1 (1.15%), O: 0 (0)	P: 251 (98.82%), A: 3 (1.18%), O: 0 (0)	P: 62 (98.41%), A: 1 (1.59%), O: 0 (0)	P: 83 (97.65%), A: 2 (2.235%), O: 0 (0%)
Missing residues	293-318	211-212, 302-318	A: 211, 302-318<br>B: 211, 303-318<br>C: 293-318<br>H: 211-299, 308-318	211-213, 293-318	302-318

Protein name	**D249N**	**D249N – Zn^{2+} form**	**D249N – Cd^{2+} form**	**D249N – Cu^{2+} form**	**D249N – Ni^{2+} form**
PDB code	6H88	6H87	6H8A	6H89	6H8D
Data collection	ESRF – ID29	ESRF – ID29	ESRF – ID29	ESRF – ID29	ESRF – ID29
Space group	$C\ 2\ 2\ 2_1$	$P\ 3_1\ 2\ 1$	$C\ 2\ 2\ 2_1$	$C\ 2\ 2\ 2_1$	$C\ 2\ 2\ 2_1$
Cell dimensions					
a, b, c (Å)	56.49, 62.83, 44.30	53.14, 53.14, 64.46	36.84, 94.66, 53.44	36.76, 94.63, 53.52	36.85, 94.33, 53.35
α, β, γ (°)	90, 90, 90	90, 90, 120	90, 90, 90	90, 90, 90	90, 90, 90
Resolution (Å)	1.50-42.01 (1.50-1.52)	1.50-46.02 (1.50-1.52)	1.80-47.33 (1.80-1.84	1.70-47.32 (1.70-1.73)	1.62-47.16 (1.62-1.64)
Rsym or Rmerge	0.038 (0.648)	0.050 (0.753)	0.145 (2.236)	0.040 (1.064)	0.033 (0.951)
I/σI	22.2 (2.2)	31.3 (2.9)	12.3 (1.8)	20.9 (1.4)	23.2 (1.6)
$CC_{1/2}$	1.000 (0.814)	1.000 (0.933)	0.997 (0.666)	0.999 (0.753)	0.999 (0.828)
Completeness (%)	99.2 (91.0)	99.8 (95.3)	100.0 (100.0)	99.7 (95.0)	99.7 (94.4)
Redundancy	6.3 (5.6)	12.6 (10.7)	12.2 (12.8)	6.4 (5.8)	6.4 (6.0)
Wavelength (Å)	0.975	0.975	0.975	0.975	0.975
No. unique reflections	12949	17417	9025	10686	12238
Refinement					
Resolution (Å)	1.50-42.01	1.50-46.02	1.80-47.33	1.70-47.32	1.62-47.16
Rwork/Rfree	18.21/22.66	18.93/22.71	19.24/23.67	19.59/24.58	17.30/21.36
No. atoms					
Protein	655	768	647	641	638
Ligand/ion	4	10	15	15	15
Water	74	81	55	51	63
B-factors					
Protein	21.21	28.31	30.25	33.21	32.02
Ligand/ion	βME-32.16	Zn^{2+}-21.14, βME-45.87, CO_2-53.56	SO_4^{2-}-33.42, Cd^{2+}-35.43, βME-63.33	SO_4^{2-}-34.79, Cu^{2+}-65.57, βME-50.32	SO_4^{2-}-32.67, Ni^{2+}-67.79, βME-83.44
Water	32.26	33.98	37.75	41.19	41.96
RMSD					
Bond lengths (Å)	0.013	0.020	0.013	0.015	0.028
Bond angles (°)	1.658	2.034	1.592	1.609	2.558
Ramachandran statistics [c]	P: 78 (97.50%), A: 2 (2.50%), O: 0 (0%)	P: 72 (98.63%), A: 1 (1.37%), O: 0 (0%)	P: 71 (98.61%), A: 1 (1.39%), O: 0 (0%)	P: 71 (98.61%), A: 1 (1.39%), O: 0 (0%)	P: 71 (98.61%), A: 1 (1.39%), O: 0 (0%)
Missing residues	295-318	302-318	211-212, 293-318	211-212, 292-318	211-213, 293-318

Protein name	**D249E – Zn^{2+} form**	**D249E – Cd^{2+} form**	**D249H – Cd^{2+} form**	**D249H – Cu^{2+} form**	**D249H-H285D**
PDB code	6H5K	6H9Q	6H84	6H83	6HA2
Data collection	ESRF – ID30B	ESRF – ID29	ESRF – ID23-1	ESRF – ID23-1	BESSY II – BL 14.1
Space group	P 3_1 2 1	C 2 2 2_1	C 2 2 2_1	C 2 2 2_1	C 2 2 2_1
Cell dimensions					
a, b, c (Å)	53.15, 53.15, 63.86	36.70, 94.52, 53.31	36.49, 94.52, 53.30	36.70, 95.07, 53.73	35.74, 94.30, 52.95
α, β, γ (°)	90, 90, 120	90, 90, 90	90, 90, 90	90, 90, 90	90, 90, 90
Resolution (Å)	1.54-46.03 (1.54-1.57)	1.50-47.26 (1.50-1.52)	2.04-47.26 (2.04-2.10)	1.50-35.60 (1.50-1.52)	1.50-47.15 (1.50-1.52)
Rsym or Rmerge	0.115 (0.237)	0.028 (0.963)	0.128 (1.423)	0.058 (1.735)	0.046 (1.346)
I/σI	20.8 (11.0)	22.3 (1.3)	13.9 (1.8)	20.1 (1.4)	18.6 (1.4)
$CC_{1/2}$	0.997 (0.990)	1.000 (0.615)	0.998 (0.603)	0.999 (0.723)	1.000 (0.582)
Completeness (%)	99.9 (98.6)	99.6 (94.1)	99.7 (95.6)	99.6 (91.6)	99.7 (94.1)
Redundancy	18.8 (18.5)	4.3 (4.2)	12.9 (10.7)	13.0 (11.5)	7.8 (7.3)
Wavelength (Å)	0.97626	0.976247	0.977999	0.977999	0.9184
No. unique reflections	15927	15228	6119	15478	14775
Refinement					
Resolution (Å)	1.54-46.03	1.50-47.26	2.04-47.26	1.50-35.60	1.50-47.15
Rwork/Rfree	14.76/18.62	19.33/21.41	18.97/22.21	19.42/22.28	16.08/19.87
No. atoms					
Protein	715	646	639	653	640
Ligand/ion	7	15	15	16	4
Water	78	72	22	47	52
B-factors					
Protein	21.29	26.93	39.60	40.61	30.60
Ligand/ion	Zn^{2+}-10.89, βME-36.12	SO_4^{2-}-31.68, Cd^{2+}-24.04, βME-72.65	SO_4^{2-}-46.19, Cd^{2+}-75.50, βME-78.26	SO_4^{2-}-29.76, Cu^{2+}-61.52, βME-49.19, Na^{+}-57.50	βME-42.57
Water	34.01	37.23	40.79	40.18	41.08
RMSD					
Bond lengths (Å)	0.011	0.017	0.016	0.013	0.015
Bond angles (°)	1.511	1.826	1.674	1.587	1.603
Ramachandran statistics [c]	P: 84 (98.82%), A: 1 (1.18%), O: 0 (0)	P: 73 (98.65%), A: 1 (1.35%), O: 0 (0)	P: 78 (98.73%), A: 1 (1.27%), O: 0 (0%)	P: 71 (98.61%), A: 1 (1.39%), O: 0 (0%)	P: 71 (98.61%), A: 1 (1.39%), O: 0 (0%)
Missing residues	293, 303-318	211-212, 293-318	293-318	211-212, 293-318	211-213, 293-318

Protein name	**H285D**	**H285D – Cd^{2+} form**	**E289H – Ni^{2+} form**	**H264E-E289H** [a]	**H264E-E289H – Cd^{2+} form**
PDB code	6H8I	6H9T	6H81	6HAN	6H85
Data collection	ESRF – ID29	ESRF – ID29	ESRF – ID23-1	BESSY II – BL 14.1	DLS – I04
Space group	C2 2 21	C2 2 21	C 2 2 2_1	P 2_1 2_1 2_1	C 2 2 2_1
Cell dimensions					
a, b, c (Å)	36.44, 94.12, 53.07	36.37, 94.56, 53.30	37.06, 94.53, 53.54	65.59, 66.64, 113.29	36.23, 94.25, 53.10
α, β, γ (°)	90, 90, 90	90, 90, 90	90, 90, 90	90, 90, 90	90, 90, 90
Resolution (Å)	1.40-47.06 (1.40-1.42)	1.70-47.28 (1.70-1.73)	1.50-47.27 (1.50-1.53)	2.60-46.75 [b] (2.60-2.71)	2.00-53.10 (2.00-2.05)
Rsym or Rmerge	0.036 (1.253)	0.040 (0.829)	0.049 (2.010)	0.15 (1.191)	0.051 (0.175)
I/σI	21.4 (1.2)	19.1 (1.6)	22.6 (1.1)	10.5 (1.4)	29.1 (8.9)
$CC_{1/2}$	0.999 (0.639)	1.000 (0.664)	0.999 (0.611)	0.997 (0.595)	0.999 (0.993)
Completeness (%)	99.9 (98.3)	99.5 (98.5)	99.5 (90.4)	99.6 (97.3)	99.6 (95.1)
Redundancy	7.2 (6.6)	4.2 (4.0)	13.2 (10.8)	8.4 (6.5)	12.4 (10.2)
Wavelength (Å)	0.976247	0.976247	0.977999	0.9184	0.9795
No. unique reflections	18410	10419	15422	15869	6470
Refinement					
Resolution (Å)	1.40-47.06	1.70-35.40	1.50-47.27	2.60-46.75	2.00-47.12
Rwork/Rfree	15.84/18.54	19.04/22.55	19.78/21.31	21.05/25.90	18.61/22.01
No. atoms					
Protein	665	659	632	A-725, B-662, C-729, D-632	628
Ligand/ion	14	15	15	53	15
Water	59	54	39	56	28
B-factors					
Protein	27.01	31.72	37.22	A-44.10, B-43.413, C-51.88, D-47.42	39.53
Ligand/ion	SO_4^{2-}-30.98, βME-49.09	SO_4^{2-}-35.85, Cd^{2+}-41.81, βME-71.72	SO_4^{2-}-37.40, Ni^{2+}-84.22, βME-74.60	SO_4^{2-}-44.88, βME-76.59, 1PE-37.31, Cl^{-}-48.61	SO_4^{2-}-46.70, Cd^{2+}-46.92, βME-64.45
Water	37.98	39.60	43.60	35.28	41.40
RMSD					
Bond lengths (Å)	0.014	0.014	0.009	0.007	0.013

Bond angles (°)	1.658	1.573	1.458	1.116	1.606
Ramachandran statistics [c]	P: 66 (98.51%), A: 1 (1.49%), O: 0 (0)	P: 71 (98.61%), A: 1 (1.39%), O: 0 (0)	P: 71 (98.61%), A: 1 (1.39%), O: 0 (0%)	P: 331 (96.50%), A: 8 (2.33%), O: 4 (1.17%)	P: 78 (98.73%), A: 1 (1.27%), O: 0 (0%)
Missing residues	211-212, 293-318	211, 293-318	211-213, 293-318	A: 211, 306-318, B: 295-318, C: 306-318, D: 293-318	211, 293-318

Protein name	**E289D – Mn^{2+} form** [a]	**E289D – Cd^{2+} form** [a]
PDB code	6H9P	6HHS
Data collection	ESRF – ID29	BESSY II – BL 14.1
Space group	P 6_2 2 2	I 1 2 1
Cell dimensions		
a, b, c (Å)	78.26, 78.26, 175.50	83.60, 94.54, 107.49
α, β, γ (°)	90, 90, 120	90.00 91.76 90.00
Resolution (Å)	2.09-44.28 [b] (2.09-2.15)	2.70-47.27 [b] (2.70-2.83)
Rsym or Rmerge	0.081 (1.762)	0.273 (1.117)
I/σI	18.5 (1.2)	5.7 (1.7)
$CC_{1/2}$	1.000 (0.837)	0.984 (0.639)
Completeness (%)	99.0 (87.6)	99.3 (97.5)
Redundancy	17.6 (13.8)	6.8 (6.9)
Wavelength (Å)	0.976247	0.9184
No. unique reflections	19491	22985
Refinement		
Resolution (Å)	2.10 - 44.28	2.70-47.27
Rwork/Rfree	21.41/23.92	24.96/29.04
No. atoms		
Protein	A-723, B-617, C-630	A-641, B-678, C-629, D-639, E-635, F-633, G-629, M-40
Ligand/ion	20	73
Water	86	113
B-factors		
Protein	A-39.93, B-43.63, C-54.34	A-36.29, B-40.00, C-33.67, D-37.23, E-39.30, F-34.58, G-46.72, M-50.86

Ligand/ion	SO_4^{2-}-36.79, Mn^{2+}-76.74, βME-58.10	SO_4^{2-}-35.78, Cd^{2+}-28.70, βME-53.39
Water	41.29	21.63
RMSD		
Bond lengths (Å)	0.017	0.009
Bond angles (°)	1.904	1.346
Ramachandran statistics [c]	P: 238 (99.58%), A: 1 (0.42%), O: 0 (0)	P: 559 (98.07%), A: 8 (1.40%), O: 3 (0.53%)
Missing residues	A- 303-318, B- 211-212, 292-318, C- 292-318	A: 211-212, 296-318 B: 211-213, 302-318 C: 211-212, 294-318 D: 294-318 E: 211, 294-318 F: 211-212, 295-318 G: 211-212, 293-318 M: unknown residues

Values in parentheses are for the highest resolution shell.

One crystal was used per dataset.

Data was collected at 100K.

[a] Collection statistics are given after the Aimless scaling[3]; data were further processed with the STARANISO server (http://staraniso.globalphasing.org/cgi-bin/staraniso.cgi; Global Phasing Ltd., Cambridge, UK).

[b] Resolution is given for best axis, as anisotropic data reduction in other axes resolution is worse.

[c] P- Preferred region, A- Allowed region, and O- outliers.

Table S3: Refinement software used for structure solution of MamM CTD constructs with different metals.

Protein name	**PDB code**	**Refmac5**[4]	**Phenix**[5]	**PDB REDO**[6]
**H264E**	6H5V	V		V
**H264E – Zn^{2+} form1**	6H5M	V		V
**H264E – Zn^{2+} form2**	6H5U	V		V
**H264E – Cd^{2+} form1**	6H8G	V		V
**H264E – Cd^{2+} form2**	6HAO		V	V
**D249N**	6H88		V	V
**D249N – Zn^{2+} form**	6H87	V	V	V
**D249N – Cd^{2+} form**	6H8A	V		V
**D249N – Cu^{2+} form**	6H89	V		V
**D249N – Ni^{2+} form**	6H8D	V		
**D249E – Zn^{2+} form**	6H5K	V		V
**D249E – Cd^{2+} form**	6H9Q	V		V
**D249H – Cd^{2+} form**	6H84	V		V
**D249H – Cu^{2+} form**	6H83	V		V
**D249H-H285D**	6HA2	V		V
**H285D**	6H8I	V		V
**H285D – Cd^{2+} form**	6H9T	V		V
**E289H – Ni^{2+} form**	6H81	V		V
**H264E-E289H**	6HAN	V	V	V
**H264E-E289H – Cd^{2+} form**	6H85	V		V
**E289D – Mn^{2+} form**	6H9P	V		V
**E289D – Cd^{2+} form**	6HHS	V	V	V

Supplementary Figures

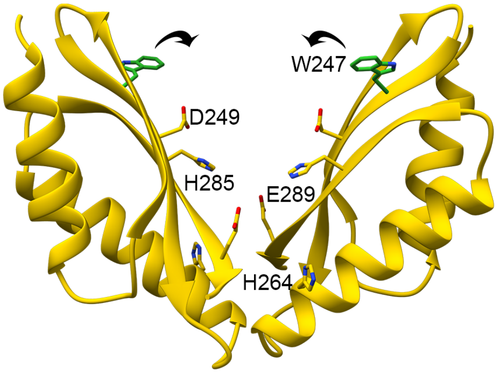

Figure S1: The dimeric apo MamM CTD WT structure (pdb code: 3W5X[7]) with binding site residues (D249, H264, H285, E289) and the fluorescence-active tryptophan residue (W247, green) indicated. Black arrows correspond to the movement of the monomers toward each other.

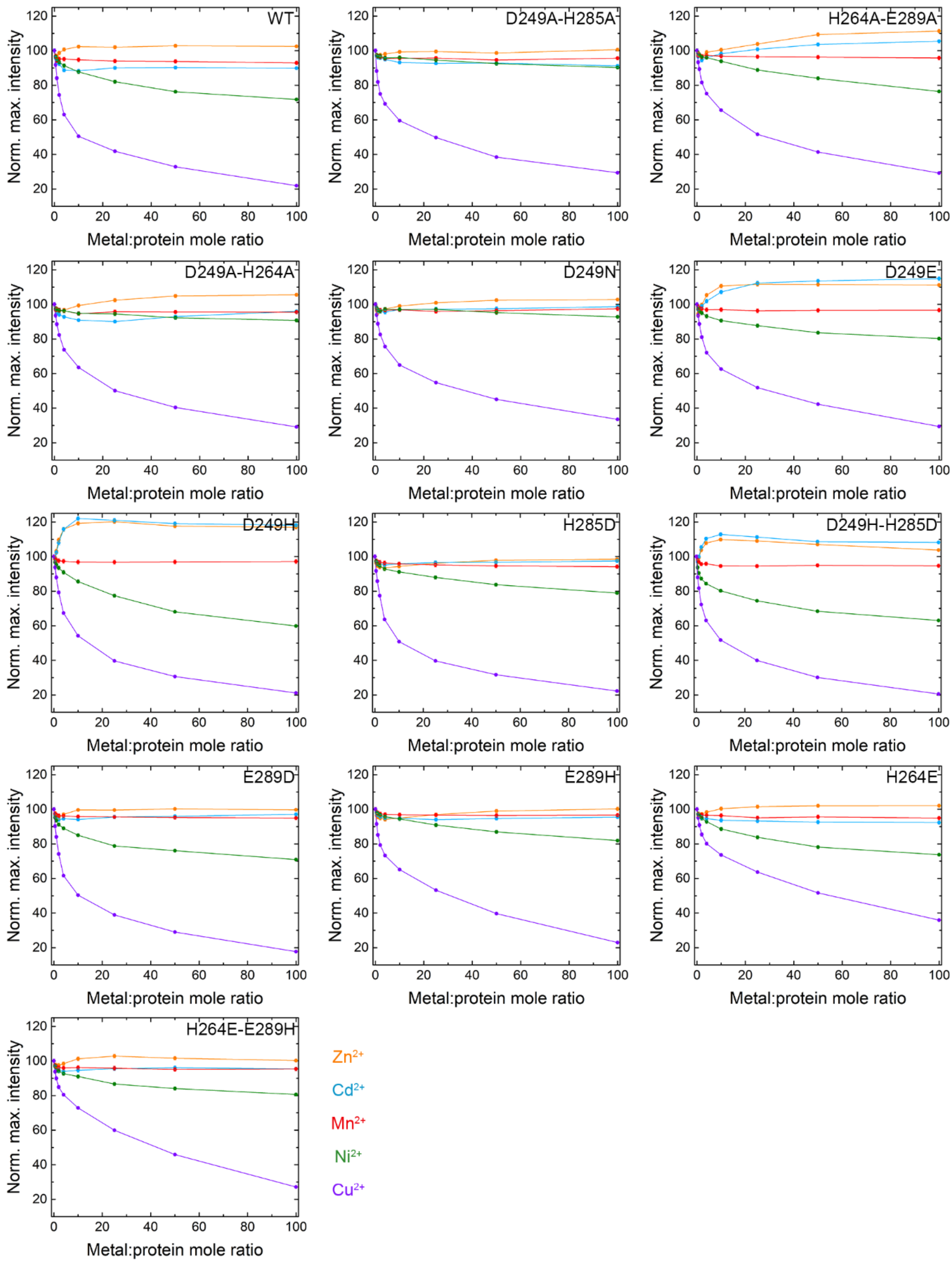

<u>Figure S2:</u> Normalized fluorescence intensity compared to no metal as function of metal:protein ratio of all MamM CTD constructs. Each graph represents one MamM CTD construct: WT (adapted from Barber-Zucker *et al.* 2020[1]), D249A-H285A, H264A-E289A, D249A-H264A, D249N, D249E, D249H, H285D, D249H-H285D, E289D, E289H, H264E and H264E-E289H. MamM CTD proteins at 5 μM

concentration were titrated using metal solutions ($ZnCl_2$, orange; $CdCl_2$, blue; $MnCl_2$, red; $NiCl_2$, green; $CuSO_4$, purple) to reach different metal:protein ratios (intensity was normalized due to the change in MamM CTD concentration) and emission spectra were recorded. Samples were measured at an excitation of λex 297 nm and the emission spectrum for each metal concentration was recorded between 310-450 nm. For each protein, the data presented is the average of three independent measurements.

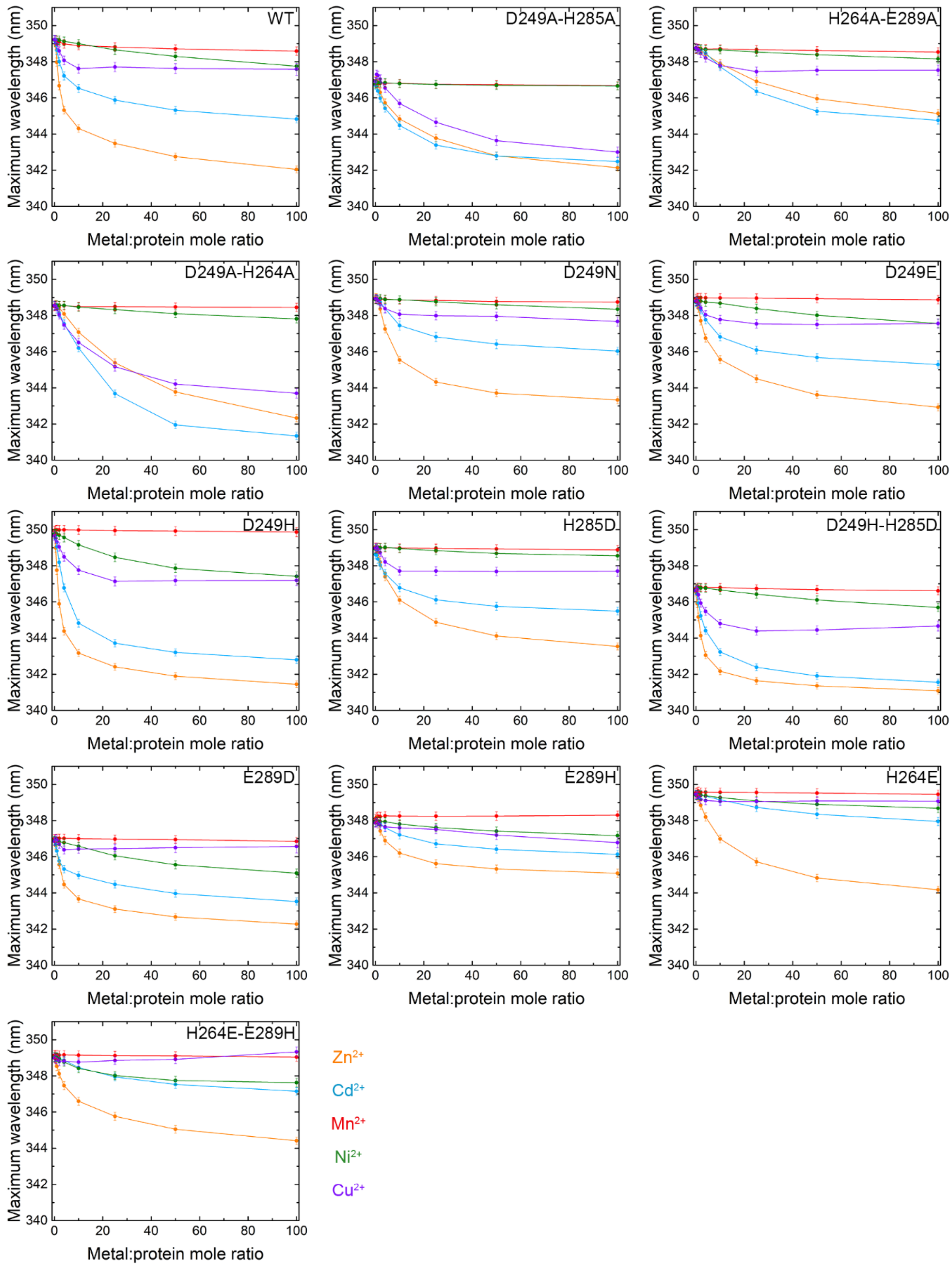

Figure S3: Maximum wavelength as function of metal:protein ratio of all MamM CTD constructs. Each graph represents one MamM CTD construct: WT (adapted from Barber-Zucker *et al.* 2020[1]), D249A-H285A, H264A-E289A, D249A-H264A, D249N, D249E, D249H, H285D, D249H-H285D, E289D, E289H, H264E and H264E-E289H. MamM CTD proteins at 5 µM concentration were titrated using

metal solutions ($ZnCl_2$, orange; $CdCl_2$, blue; $MnCl_2$, red; $NiCl_2$, green; $CuSO_4$, purple) to reach different metal:protein ratios (intensity was normalized due to the change in MamM CTD concentration) and emission spectra were recorded. Samples were measured at an excitation of λex 297 nm and the emission spectrum for each metal concentration was recorded between 310-450 nm. For each protein, the data presented is the average of three independent measurements.

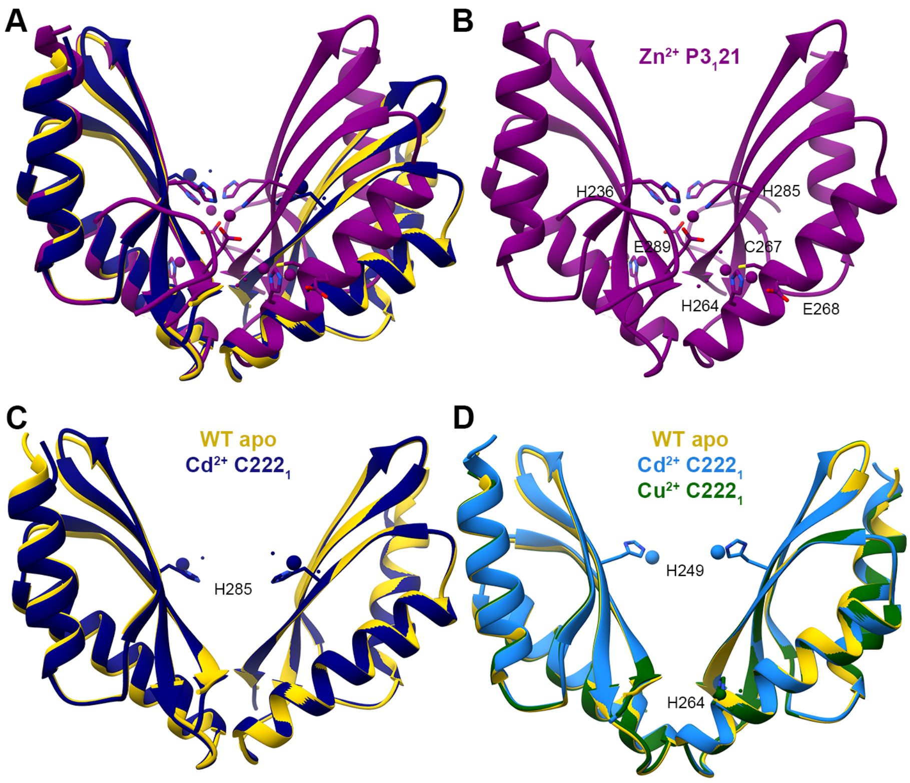

Figure S4: Crystal structures of MamM CTD D249E and D249H mutants with different metals. (A) Crystal structures of MamM CTD D249E with Cd^{2+} (SG C222$_1$, navy blue, pdb code: 6H9Q) and Zn^{2+} (SG P3$_1$21, dark magenta, pdb code: 6H5K) overlaid onto apo MamM CTD WT structure (SG C222$_1$, gold, pdb code: 3W5X[7]). (B) Crystal structure of MamM CTD D249E with Zn^{2+} exhibits the same Zn^{2+} binding sites and conformation as that of D249N with Zn^{2+}. (C) Crystal structures of MamM CTD D249E with Cd^{2+} (SG C222$_1$, navy blue) show the same conformation as apo MamM CTD WT (SG C222$_1$, gold). Cd^{2+} is bound by H285, water molecules and H213 and E215 from a non-biological monomer from an adjacent unit (not shown). (D) Crystal structures of MamM CTD D249H with Cd^{2+} (SG C222$_1$, dodger blue, pdb code: 6H84) and Cu^{2+} (SG C222$_1$, dark green, pdb code: 6H83) overlaid onto apo MamM CTD WT structure (SG C222$_1$, gold, pdb code: 3W5X[7]). While Cu^{2+} is chelated by H264 and water molecules, Cd^{2+} is chelated by H249 and E215 from a non-biological monomer from an adjacent unit (not shown).

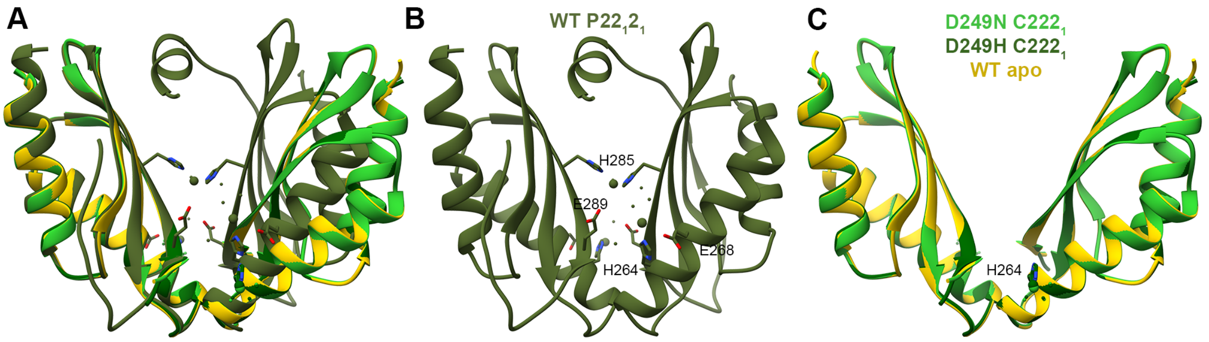

Figure S5: Crystal structures of different MamM CTD constructs bound to Cu^{2+}. (A) Crystal structures of MamM CTD WT with Cu^{2+} (SG P22$_1$2$_1$, dark olive green, pdb code: 6GP6[1]), MamM CTD D249N with Cu^{2+} (SG C222$_1$, lime green, pdb code: 6H89) and MamM CTD D249H with Cu^{2+} (SG C222$_1$, dark green, pdb code: 6H83) overlaid onto apo MamM CTD WT structure (SG C2221, gold, pdb code: 3W5X[7]). (B) The crystal structure of MamM CTD WT with Cu^{2+} exhibits tight conformation and contains one central Cu^{2+} binding site and two symmetrical peripheral binding sites. (C) Crystal structures of MamM CTD D249N with Cu^{2+} (SG C222$_1$, lime green) and MamM CTD D249H with Cu^{2+} (SG C222$_1$, dark green) are bound only by H264 and water molecules and show the same conformation as apo MamM CTD WT (SG C222$_1$, gold).

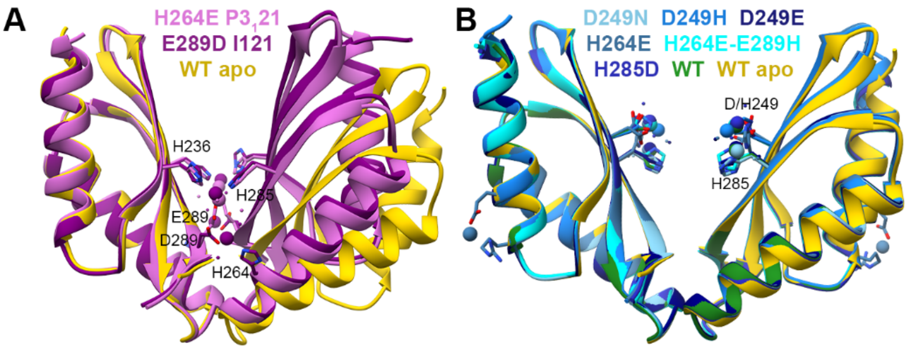

Figure S6: Crystal structures of different MamM CTD constructs bound to Cd^{2+}. (A) Crystal structures of MamM CTD H264E with Cd^{2+} (SG $P3_121$, orchid, pdb code: 6HAO) and MamM CTD E289D with Cd^{2+} (SG I121, dark magenta, pdb code: 6HHS) overlaid onto apo MamM CTD WT structure (SG C2221, gold, pdb code: 3W5X[7]). Both structures exhibit similar conformation tighter than the WT apo, however the number of bound Cd^{2+} ions per dimer and the binding sites differ between the two mutants (for E289D, two residues from a third monomer also participate in the chelation of each Cd^{2+}-pair, see Figure 3). (B) Crystal structures of MamM CTD with Cd^{2+}: H264E (steel blue, pdb code: 6H8G), H264E-E289H (cyan, pdb code: 6H85), D249N (sky blue, pdb code: 6H8A), D249E (navy blue, pdb code: 6H9Q), D249H (dodger blue, pdb code: 6H84), H285D (medium blue, pdb code: 6H9T) and WT (forest green, pdb code: 6GMT[1]) (all structures in SG $C222_1$) overlaid onto apo MamM CTD WT structure (SG C2221, gold, pdb code: 3W5X[7]). Although the number of bound Cd^{2+} cations bound (one or two per monomer) and the exact residues that participate in the binding at each MamM CTD constructs differ, all structures show the same conformation and share similar binding properties (in most of the constructs, the Cd^{2+} cations are also chelated by H213 and/or E215 from a non-biological monomer from an adjacent unit (not shown)).

Supplementary References

1. Barber-Zucker, S. *et al.* The cation diffusion facilitator protein MamM's cytoplasmic domain exhibits metal-type dependent binding modes and discriminates against Mn^{2+}. Preprint at *bioRxiv***,** DOI: 10.1101/2020.02.02.930644 (2020).

2. Barber-Zucker, S., Shaanan, B. & Zarivach, R. Transition metal binding selectivity in proteins and its correlation with the phylogenomic classification of the cation diffusion facilitator protein family. *Sci. Rep.* **7**, 16381 (2017).

3. Evans, P. R. & Murshudov, G. N. How good are my data and what is the resolution? *Acta Crystallogr. Sect. D Biol. Crystallogr.* **69**, 1204–1214 (2013).

4. Murshudov, G. N. *et al.* REFMAC5 for the refinement of macromolecular crystal structures. *Acta Crystallogr. D. Biol. Crystallogr.* **67**, 355–367 (2011).

5. Adams, P. D. *et al. PHENIX*: a comprehensive Python-based system for macromolecular structure solution. *Acta Crystallogr. Sect. D Biol. Crystallogr.* **66**, 213–221 (2010).

6. Joosten, R. P., Long, F., Murshudov, G. N. & Perrakis, A. The PDB_REDO server for macromolecular structure model optimization. *IUCrJ* **1**, 213–220 (2014).

7. Zeytuni, N. *et al.* Cation diffusion facilitators transport initiation and regulation is mediated by cation induced conformational changes of the cytoplasmic domain. *PLoS One* **9**, e92141 (2014).
